# Photo-modulation of supramolecular polymorphism in the self-assembly of a scissor-shaped azobenzene dyad into nanotoroids and fibers†

**DOI:** 10.1039/d2sc00690a

**Published:** 2022-02-23

**Authors:** Natsuki Suda, Takuho Saito, Hironari Arima, Shiki Yagai

**Affiliations:** Division of Advanced Science and Engineering, Graduate School of Science and Engineering, Chiba University 1-33 Yayoi-cho, Inage-ku Chiba 263-8522 Japan; Department of Applied Chemistry and Biotechnology, Graduate School of Engineering, Chiba University 1-33 Yayoi-cho, Inage-ku Chiba 263-8522 Japan yagai@faculty.chiba-u.jp; Institute for Global Prominent Research (IGPR), Chiba University 1-33 Yayoi-cho, Inage-ku Chiba 263-8522 Japan

## Abstract

Recent advances in the research field of supramolecularly engineered dye aggregates have enabled the design of simple one-dimensional stacks such as fibers and of closed structures such as nanotoroids (nanorings). More complex and advanced supramolecular systems could potentially be designed using a molecule that is able to provide either of these distinct nanostructures under different conditions. In this study, we introduced bulky but strongly aggregating cholesterol units to a scissor-shaped azobenzene dyad framework, which affords either nanotoroids, nanotubes, or 1D fibers, depending on the substituents. This new dyad with two *trans*-azobenzene arms shows supramolecular polymorphism in its temperature-controlled self-assembly, leading to not only oligomeric nanotoroids as kinetic products, but also to one-dimensional fibers as thermodynamic products. This supramolecular polymorphism can also be achieved *via* photo-triggered self-assembly, *i.e.*, irradiation of a monomeric solution of the dyad with two *cis*-azobenzene arms using strong visible light leads to the preferential formation of nanotoroids, whereas irradiation with weak visible light leads to the predominant formation of 1D fibers. This is the first example of a successful light-induced modulation of supramolecular polymorphism to produce distinctly nanostructured aggregates under isothermal conditions.

## Abstract 2

Introduction of the bulky yet strongly aggregating cholesterol units to an azobenzene dyad lead to a supramolecular polymorphism not only in its temperature-controlled but also in photo-triggered self-assembly, leading to toroids and helical fibers.

## Graphical abstract

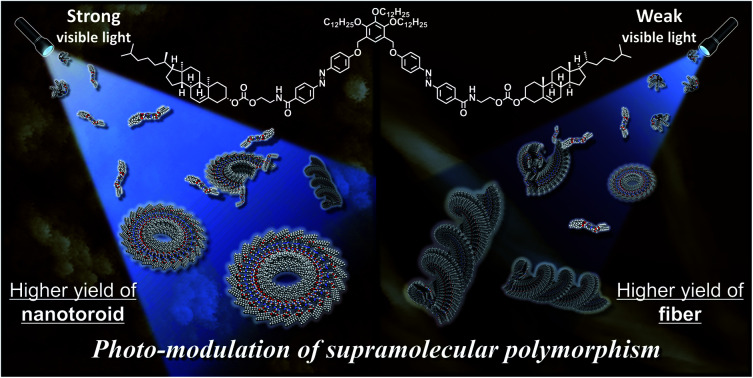

## Introduction

Recent advances in the area of molecular self-assembly have allowed creating supramolecularly engineered nanostructures from various functional molecules.^1^ Based on the extensive knowledge regarding the specificity and selectivity of supramolecular interactions to connect molecules as well as preorganize them, one can design not only one-dimensional nanofibers and sheets^2^ but also sophisticated “closed” nanostructures such as rings and tubes.^3^ The former one-dimensional nanofibers can be categorized as supramolecular polymers, and, like their covalent counterparts, they exhibit attractive properties such as gelation and liquid crystallinity in the bulk.^2^ On the other hand, the latter, materials with a closed nanostructure are expected to show interesting properties at the single-nanostructure level.^3^ Accordingly, molecules that could form either one-dimensional or closed nanostructures depending on conditions would allow the establishment of single-component complex supramolecular systems whose physical properties and functions could be switched *via* pathway control through external stimuli. In this context, we have developed a strategy in this study to use recently reported azobenzene dyad molecules to impart the ability to form not only discrete toroidal nanoassemblies (nanotoroids), but also one-dimensional nanofibers.

**Fig. 1:**
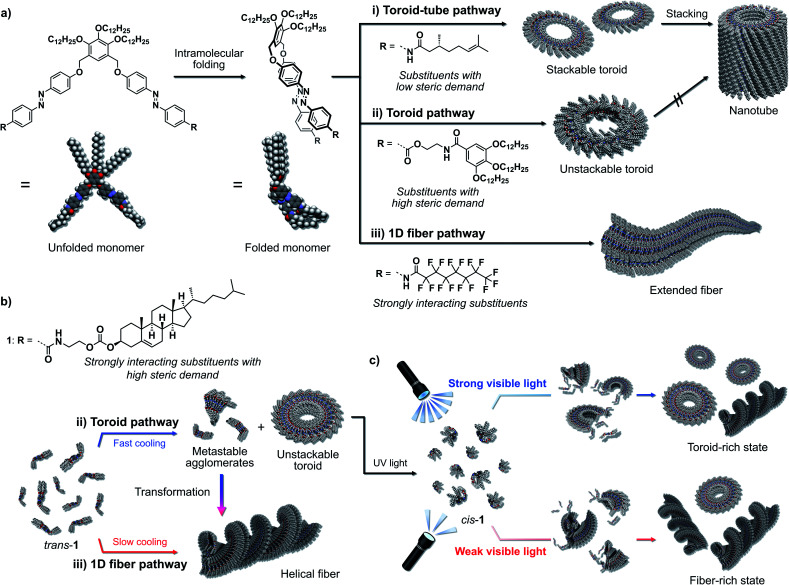
(a) The basic molecular structure of the scissor-shaped azobenzene dyad. (i)–(iii) Schematic representation of the substituent-dependent self-assembly pathways of the dyad into (i) stackable nanotoroids and nanotubes, (ii) unstackable nanotoroids, or (iii) extended fibers. (b) Thermoregulated and (c) photo-regulated self-assembly pathways of 1 into nanotoroids and helical fibers.

We have explored the self-assembly and photoresponsivity properties of a series of scissor-shaped azobenzene dyads,^4^ in which azobenzene units with various substituents on their amide groups are dimerized *via* a 3,4,5-(tridodecyloxy)xylylene moiety (Fig. 1).^5^ In nonpolar solvents, these dyads can adopt “wedge”-shaped folded structures through intramolecular hydrogen bonding and π–π stacking interactions. As a result of their unique folded structures, these dyads self-assemble to form nanotoroids. When substituents with low steric demand are introduced as the R groups in Fig. 1a, the resulting nanotoroids undergo further hierarchical stacking in solution upon cooling to form tubular nanostructures (nanotubes; Fig. 1a-i).^6^ On the other hand, we have recently discovered that the introduction of substituents with high steric demand through flexible linkers results in the formation of discrete nanotoroids that do not stack due to the deteriorated planarity of the nanotoroids (Fig. 1a-ii).^7^ In another study, we introduced strongly interacting perfluoroalkyl chains instead of the original alkyl chains. In that case, the dyad shows a distinct (non-toroidal) self-assembly pathway to afford gel-forming one-dimensional fibers due to fluorophilic interactions (Fig. 1a-iii).^8^

Based on the above monomer structure/self-assembly relationships, we envisaged that the introduction of bulky but strongly aggregating substituents might realize supramolecular polymorphism^9^*via* a monomer that could provide both discrete toroidal and extended fibrous nanoaggregates. As one such substituent, we herein introduced cholesterol units^10^ and investigated the self-assembly of the resulting dyad 1 (Fig. 1b). This new dyad kinetically self-assembled to form nanotoroids, whereas it thermodynamically assembled into one-dimensional fibers in temperature-regulated supramolecular polymerization. We also demonstrate that the nanotoroids are kinetically stable species due to their closed structures, which dissociate into monomers upon irradiation with UV light. Importantly, during reassembly triggered by visible light, the nanotoroid/fiber ratio was found to depend on the intensity of the visible light, which suggests the possibility to control the pathway using light (Fig. 1c).

## Results and discussion

### Kinetic self-assembly

Cholesterol-functionalized azobenzene dyad 1 was synthesized and characterized using ^1^H and ^13^C NMR spectroscopy as well as APCI mass spectrometry (Scheme S1†). When a hot MCH solution of 1 (total concentration of the monomer, *c*_t_ = 100 μM) was rapidly cooled (quenched) from 80 to 20 °C using an ice-water bath, the π–π* absorption band of the *trans*-azobenzene moieties shifted from 346 nm to 337 nm (Fig. S1a†). In the circular dichroism (CD) spectrum, a weak bisignate CD signal with a positive maximum at 331 nm and a negative maximum at 355 nm was observed at 20 °C, whilst the molecular dissolved solution at 80 °C was CD-silent (Fig. S1b†). A comparison of the FT-IR spectra of this MCH solution and a monomeric solution of 1 in CHCl_3_ revealed that the amide groups form hydrogen bonds in MCH (Fig. S2†).

**Fig. 2:**
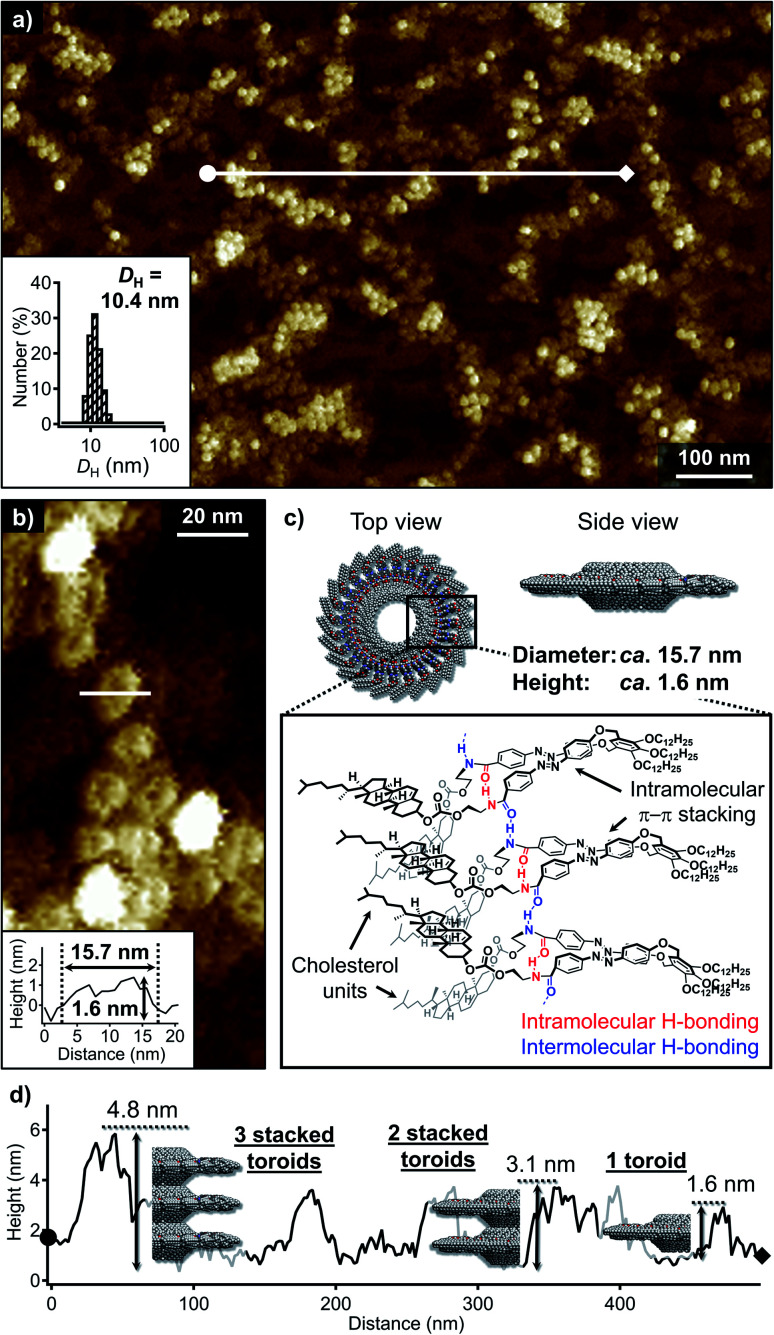
(a) and (b) AFM images of aggregates of 1 obtained by quenching a hot MCH solution (*c*_t_ = 100 μM) from 80 to 20 °C. The inset in (a) shows the DLS-derived size distribution of the MCH solution, while that in (b) shows the cross-sectional analysis along the white line. (c) Schematic representation of the proposed packing structure of 1 in a nanotoroid. (d) Cross-sectional analysis along the white line in (a) and schematic representation of the stacking of the nanotoroids.

Dynamic light scattering (DLS) measurements of the above quenched MCH solution showed a narrow size distribution centered at a hydrodynamic diameter (*D*_H_) of 10.4 nm (inset in Fig. 2a). Consistent with this result, atomic force microscopy (AFM) imaging of spin-coated samples on highly oriented pyrolytic graphite (HOPG) showed a high number of particulate nanoaggregates (Fig. 2a). A close examination of the magnified AFM images revealed a dent in the center of these particles, suggesting that they are toroidal in shape (Fig. 2b). The edge-to-edge diameter and height (thickness) of the toroidal aggregates were *ca.* 15.7 and 1.6 nm, respectively. The diameter was in good accordance with those of previously reported nanotoroids,^6*a*,*c*,7^ which suggests that the present cholesterol derivative underwent the same self-assembly process through intramolecular folding followed by curved aggregation. In contrast, their height was appreciably greater than that of nanotoroids formed by previously reported dyads with less sterically demanding amide substituents (Fig. 1a-i, *ca.* 1.0 nm). The thicker structure, along with the ambiguity of the central pore in AFM imaging clearly demonstrated that the bulky cholesterol units are located inside the nanotoroid (Fig. 2c).

The AFM image in Fig. 2a displays another unique feature of the toroidal aggregates of 1. In some places, the nanotoroids evidently exhibit different thickness, which suggests that they tend to stack on top of each other. A cross-sectional analysis revealed heights of 1.6, 3.1, and 4.8 nm (Fig. 2d). These thickness values indicate the stacking of two to three nanotoroids.^11^ Despite this higher-order-aggregation tendency, extended stacking of the nanotoroids to afford tubular assemblies, which was observed for the planar nanotoroids of previously reported dyads, was not observed even upon cooling the solution to 0 °C (Fig. S3a†). This was corroborated by the absence of significant changes in the CD spectra and DLS profiles recorded at 20 and 0 °C (Fig. S3b and c†). These observations suggest that the steric demand of the cholesterol moiety prevents the continuous stacking of the nanotoroids, and that they instead form discrete oligomeric stacks through van der Waals interactions between cholesterol moieties. This behavior is similar to anti-cooperative supramolecular polymerization by attenuated growth of supramolecular stacks.^12^

**Fig. 3:**
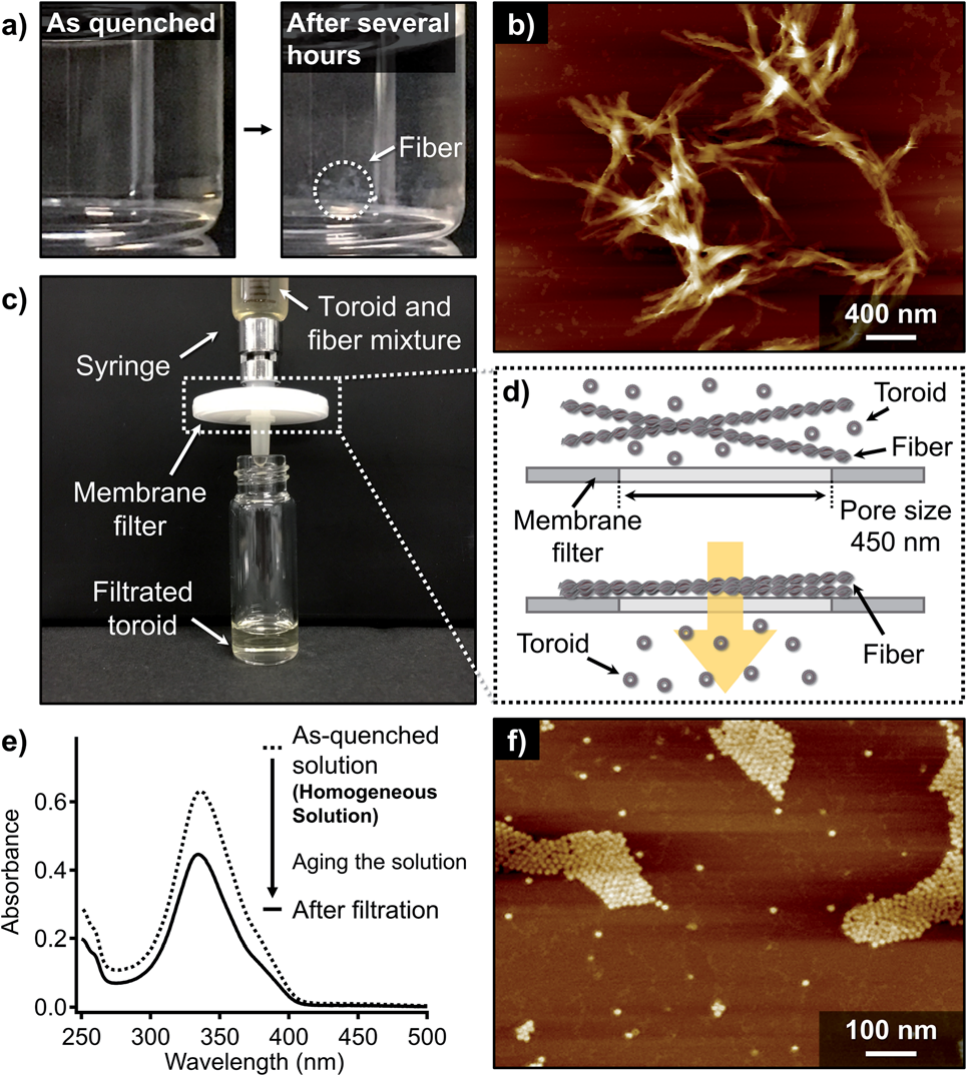
(a) Photographs of the as-quenched and precipitated MCH solution of 1 (*c*_t_ = 100 μM). (b) and (f) AFM images of (b) fibrous aggregates of 1 obtained by aging the quenched solution (*c*_t_ = 100 μM) for several hours and (f) toroidal aggregates of 1 found in the filtrate after passing through a membrane filter. (c) Photograph showing the filtration experiment to isolate nanotoroids from fibers using a membrane filter. (d) Schematic illustration of the selective passage of nanotoroids through a pore of the membrane filter. (e) UV/Vis absorption spectra of MCH solution of 1 upon quenching a hot MCH solution (*c*_t_ = 100 μM, dashed line) followed by filtration of the aged solution (black line). Based on the decrease in the absorbance, the total concentration after filtration was estimated to be 72 μM.

When the aforementioned nanotoroid solution was kept at 20 °C for several hours, precipitation was observed (Fig. 3a). AFM imaging of the precipitates visualized entangled fibrous aggregates (Fig. 3b and S4†). AFM measurements suggested a height of approximately 2.2 nm for the thinnest part of the fibers, which excludes the possibility of polymeric stacking of the nanotoroids. Together with these elongated fibers, nanotoroids were always observed in specimens prepared under these conditions, even upon aging the solutions for several days. Accordingly, under kinetic control, 1 shows closed and open self-assembly pathways leading to nanotoroids and fibers, respectively. To quantify the molar distribution of 1 into these aggregates, we attempted to separate them by passing the precipitated solution through a membrane filter with a pore size of 450 nm (Fig. 3c and d). This procedure completely removed the fibrous aggregates, and the filtrate contained only the nanotoroids, which was confirmed by AFM (Fig. 3f and S5†). Absorption measurements of the filtrate revealed that 72% of the monomers (*c*_t_ = 72 μM) aggregated as nanotoroids (Fig. 3e). Assuming that monomers can be quantitatively aggregated without being trapped in unknown aggregated states, 28% of the monomers is estimated to aggregate as fibers. Importantly, the isolated nanotoroids remained stable over several weeks without forming any precipitates, demonstrating that these nanotoroids are kinetically trapped, off-pathway aggregates^13^ in the formation of thermodynamically stable fibers.^14^

Importantly, the isolated nanotoroid solution was CD-silent (Fig. S6†) despite the weak Cotton effect observed for the as-quenched solution described above (Fig. S1b†). On one hand, this observation demonstrates that the molecular chirality of the cholesterol units does not influence the toroidal assembly. On the other hand, the initially observed weak CD signal (Fig. S1b†) can be attributed to a metastable species that is eventually transformed into the thermodynamically stable fibrous aggregates. Such a metastable state could occur, for example, *via* intramolecular hydrogen bonding between amide groups to produce a seven-membered intramolecularly H-bonded pseudocycle.^7,15^

### Thermodynamic self-assembly

**Fig. 4:**
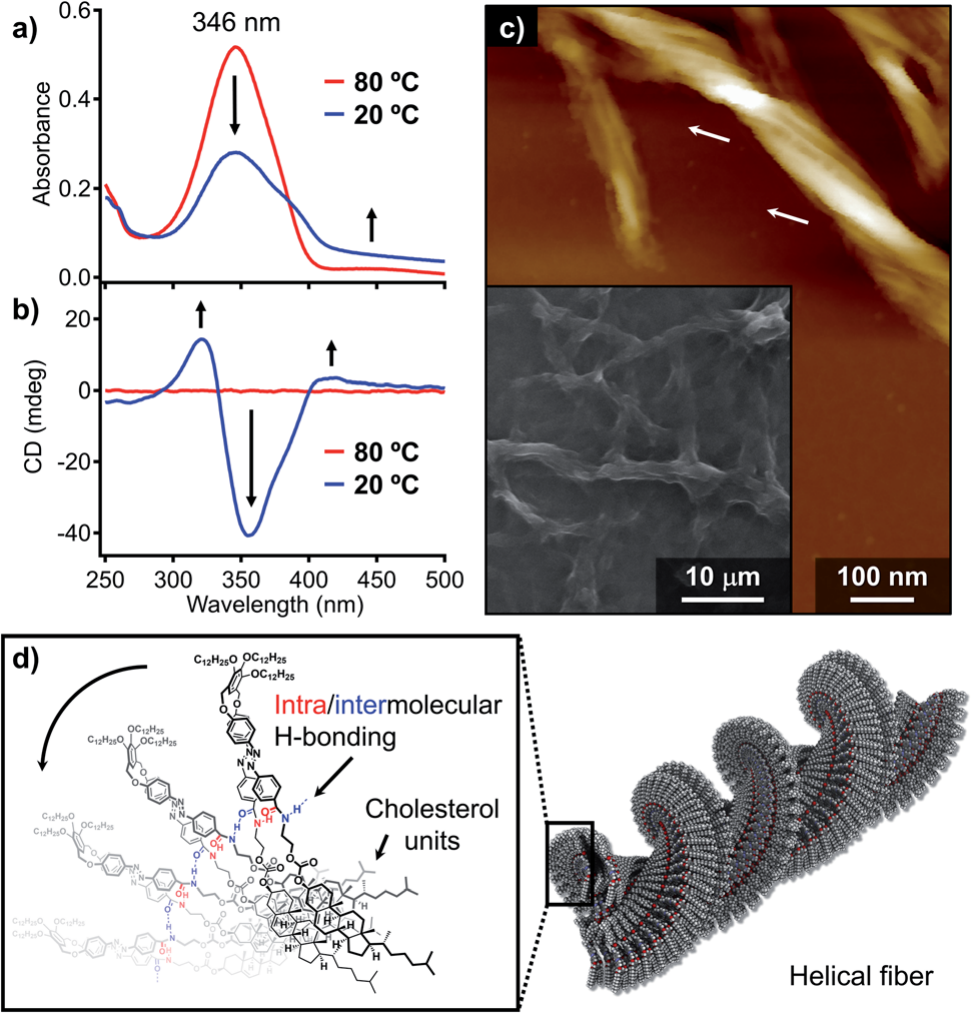
(a) UV/Vis absorption and (b) CD spectra of 1 in MCH (*c*_t_ = 100 μM) at 80 °C (red lines) and 20 °C (blue lines) upon cooling at a rate of 1 °C min^−1^ (c) AFM image of left-handed superhelical fibers of 1. The white arrows indicate the left-handed superhelicity. The inset shows a SEM image of precipitated fibers. (d) Schematic representation of the proposed packing structure of 1 in fiber.

To further investigate the above supramolecular polymorphism, self-assembly of 1 was conducted using a protocol whereby more thermodynamically favorable products can be formed. For this purpose, a hot MCH solution (*c*_t_ = 100 μM) was cooled from 80 to 20 °C at a rate of 1 °C min^−1^ using a Peltier temperature-control unit. During cooling, precipitation of the fibrous aggregates was already observed at around 45 °C by a monotonous decrease in the absorption spectrum with an increase in the baseline (Fig. 4a and S7†). The suspension showed pronounced CD signals (Fig. 4b), whose shape and intensity were distinctively different from those of the quenched solution (Fig. S1b†). The yield of the fibrous aggregates thus obtained was estimated by the above-described filtration protocol to be 96% based on the monomer (Fig. S8†).

With a significant amount of the fibers obtained from applying slow cooling in hand, we further investigated the detailed morphological features of the fibers. Scanning electron microscopy (SEM) images showed entangled fibers of hundreds of micrometers in length (inset in Fig. 4c), while AFM images revealed multistranded helical structures for these fibers (Fig. 4c). A detailed investigation of the helical structures *via* AFM confirmed that these exhibit exclusively left-handed superhelicity (Fig. S9†), indicating that the molecular chirality of the cholesterol moieties can be transferred to the macroscopic helicity.^16^ Powder X-ray diffraction (XRD) analysis of the fibrous precipitates showed diffractions that arise from a 2D rectangular columnar lattice with lattice parameters of *a* = 6.27 and *b* = 2.53 nm (Fig. S10†). Accordingly, we would like to propose a one-dimensional helical columnar organization for the folded molecules as the thermodynamic aggregation pathway of 1 (Fig. 4d). Importantly, an FT-IR analysis showed that the amide groups in the fibrous aggregates hydrogen-bonded (*ν*_NH_ = 3285 cm^−1^) even more strongly than in the nanotoroids (*ν*_NH_ = 3314 cm^−1^; Fig. S2†), which highlights that the formation of nanotoroids is a kinetic process^14*b*^ in which even weak (frustrated) intra/intermolecular hydrogen bonds can lead to aggregation with the help of a supramolecular ring-closure reaction.

### Photo-modulation of the self-assembly pathways

**Fig. 5:**
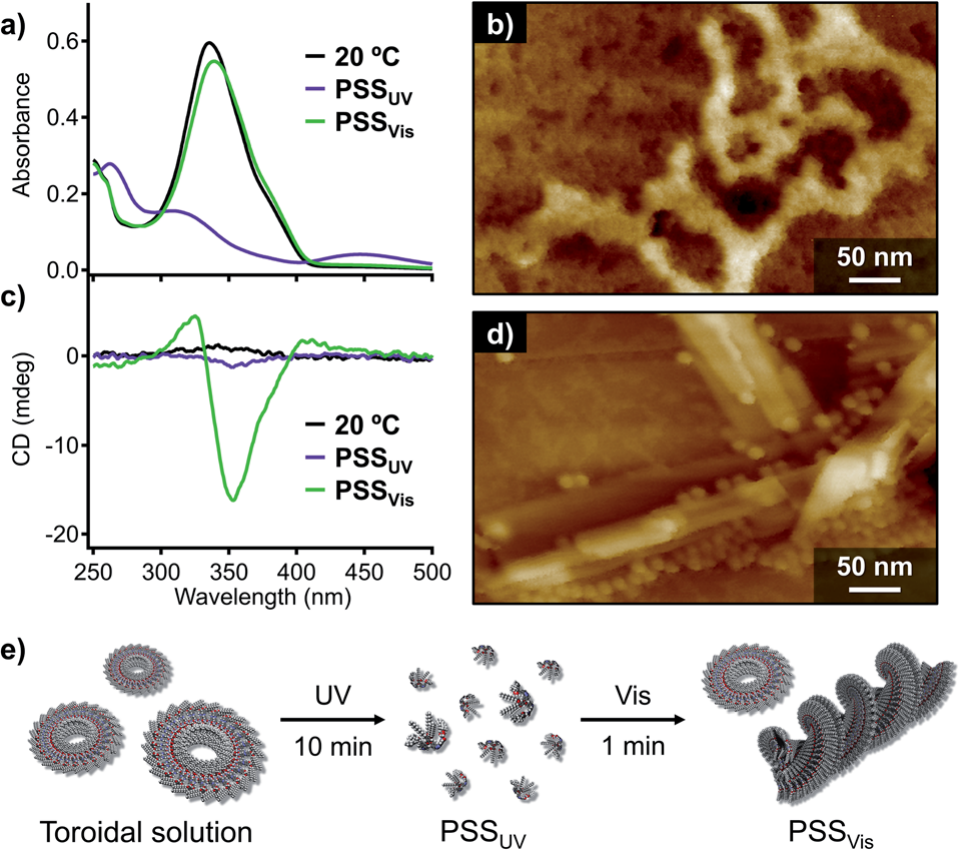
(a) UV/Vis absorption and (c) CD spectra of nanotoroids of 1 in MCH (*c*_t_ = 100 μM, black lines) after irradiation with UV light 10 min (purple lines) or visible light for 1 min (49 mW cm^−2^; green lines) at 20 °C. (b) and (d) AFM images of (b) an amorphous film of 1 and (d) a mixture of nanotoroids and fibers of 1 obtained from a MCH solution after irradiation with UV or visible light (49 mW cm^−2^). (e) Protocol for the UV- and visible-light irradiation experiment.

Having unravelled the supramolecular polymorphism of 1, we then investigated the effect of photoisomerization. Given that irradiation of the fiber suspension with UV light (*λ* = 365 nm, 30 mW cm^−2^) did not result in photoisomerization due to its inhomogeneity, we applied UV irradiation to the homogeneous toroidal solution obtained by the filtration. UV-irradiation of this nanotoroid solution (*c*_t_ = 100 μM) at 20 °C resulted in a decrease of the π–π* absorption band of the *trans*-azobenzene units and an increase of the n–π* absorption band of *cis*-azobenzene at around 450 nm (Fig. 5a). A photostationary state (PSS_UV_) was achieved after 10 min of UV irradiation, for which the *trans* : *cis* ratio (12 : 88) was estimated based on UV/Vis and ^1^H NMR control experiments (Fig. S11†). AFM imaging of the PSS_UV_ sample showed that the nanotoroids were completely converted to amorphous agglomerates due to the bent conformation of the *cis*-azobenzene units (Fig. 5b).^17^

Subsequent irradiation of the PSS_UV_ solution with visible light (*λ* = 470 nm, 49 mW cm^−2^) for 1 min induced significant *cis-*to*-trans* isomerization, as confirmed by the recovery of the absorption band of the *trans-*azobenzene units at around 339 nm, and another photostationary state (PSS_Vis_) with a *trans* : *cis* ratio of 89 : 11 was observed (Fig. 5a). Interestingly, the PSS_Vis_ solution exhibited a CD signal characteristic of fibrous helical aggregates although the CD intensity was weaker than that of the slowly cooled solution (Fig. 4b and 5c). AFM images of the PSS_Vis_ sample revealed the formation of nanotoroids as well as fibrous aggregates (Fig. 5d), which demonstrates that the supramolecular polymorphism of 1 also occurs in this photo-triggered self-assembly (Fig. 5e), albeit that unlike the self-assembly by rapid cooling, it does not go through a metastable state. Aging the PSS_Vis_ solution for several minutes resulted in the precipitation of fibrous aggregates, and the yield of the nanotoroids and fibrous aggregates based on monomer was determined to be 45% and 55%, respectively. This moderate yield of the thermodynamic products, in comparison with the aforementioned 96% yield with the 1 °C min^−1^ cooling, indicates that the self-assembly of 1 with two *trans*-azobenzene units generated at 20 °C by visible light is under kinetic control due to the rapid increase of its concentration in 1 min.^18^ Indeed, by slowly increasing its concentration through thermal back-isomerization over several days, we obtained the thermodynamically stable fibrous aggregates in 88% yield.

**Fig. 6:**
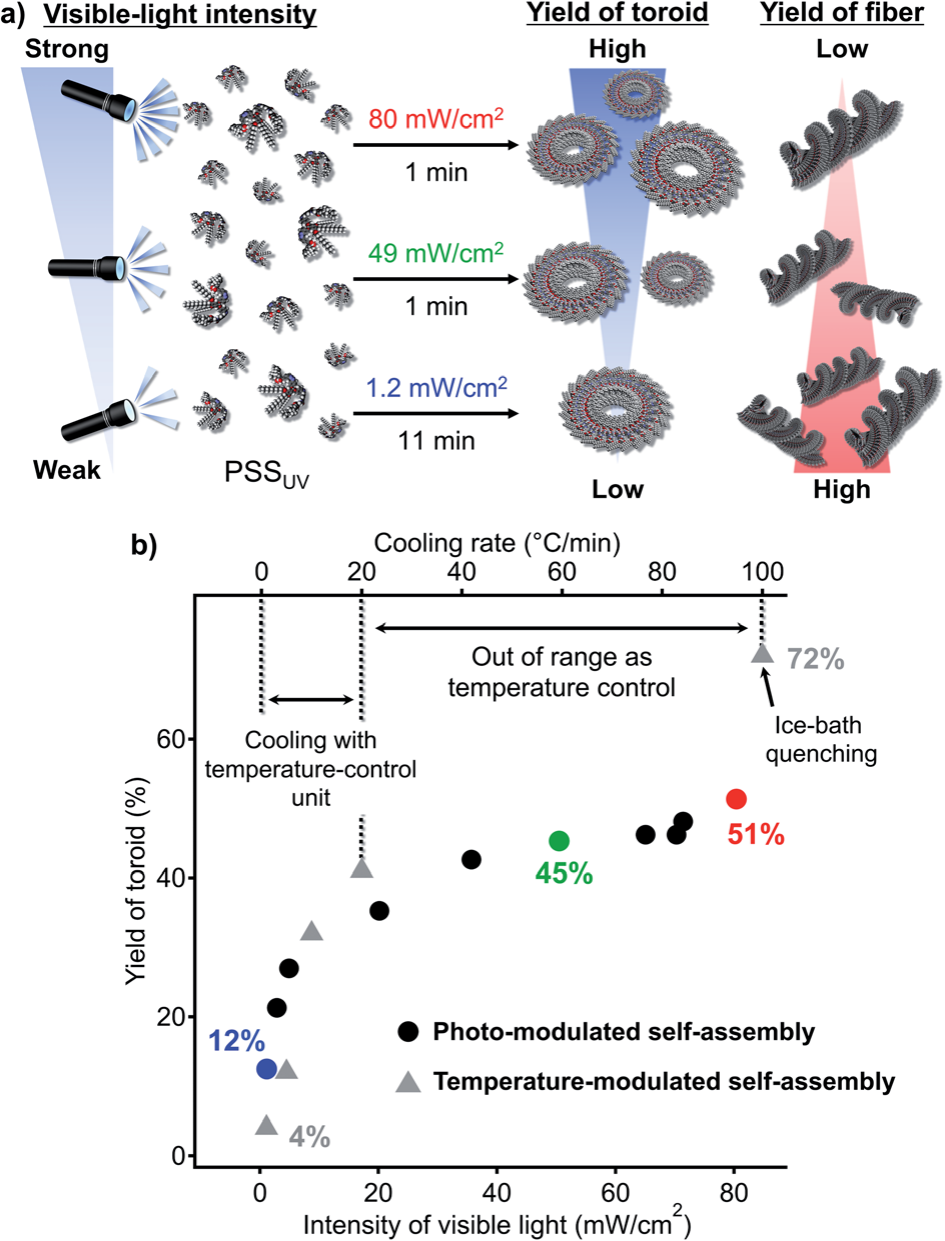
(a) Schematic representation for the photo-modulation of the nanotoroid/fiber ratio by varying the intensity of the visible light. (b) Plot of the yield of toroidal aggregates as a function of the intensity of the visible light and the cooling rate.

Given the aforementioned results, we attempted photo-modulation of the nanotoroid/fiber ratio by varying the intensity of the visible light (Fig. 6a). When we applied strong visible light (80 mW cm^−2^, 1 min) to the PSS_UV_ solution, we obtained nanotoroids in 51% yield at the PSS_Vis_ with a *trans* : *cis* ratio of 97 : 3 (red dot, Fig. 6b). This yield was higher than the 45% yield obtained using an intensity of 49 mW cm^−2^ (green dot, Fig. 6b). In contrast, by applying very weak visible light (1.2 mW cm^−2^, 11 min) to the PSS_UV_ solution, the yield of nanotoroids decreased to 12% at the corresponding PSS_Vis_ with a *trans* : *cis* ratio of 80 : 20 (blue dot, Fig. 6b). By applying varying visible-light intensities (1.2–80 mW cm^−2^), we were thus clearly able to control the nanotoroid/fiber ratio (Fig. 6b). Although the range of yields achieved using our light-modulated kinetic pathway fell within the range covered by the cooling protocol using the Peltier temperature-control unit (1–20 °C min^−1^) or ice-bath quenching (*ca.* 100 °C min^−1^), as shown in Fig. 6b (grey triangles), it is noteworthy that it is feasible to expect that more precise and finer modulation could be conveniently achieved using light. In particular, in situations in which it is necessary to obtain a moderate yield of the kinetic product using a fast cooling rate (>20 °C min^−1^), the use of strong light could present a valuable solution for this specific regime.

Finally, one may remember that the nanofibers suspended in MCH were inert to UV-irradiation, as described at the beginning of this section. Accordingly, when we irradiate a nanotoroid/fiber mixture with UV and visible light repeatedly, we can finely increase the ratio of fibers. For example, when the pure toroid solution was repeatedly irradiated with UV (30 mW cm^−2^) and visible light (49 mW cm^−2^) six times, we could increase fiber ratio from 55% for the single irradiation to 65%.

## Conclusions

Previously, we had controlled the self-assembly pathways of our azobenzene dyads through side-chain engineering (Fig. 1a). In the present study, we have introduced bulky but strongly aggregating cholesterol units to the dyad backbone, which resulted in supramolecular polymorphism to afford kinetically trapped toroidal and thermodynamically stable fibrous aggregates. The formation of the two polymorphic aggregates co-occurs and these aggregates can co-exist; their yields can be controlled by exerting kinetic control over the self-assembly pathways *via* changing the cooling rate. More importantly, we have demonstrated that this supramolecular polymorphism can be precisely and conveniently modulated under isothermal conditions by changing the intensity of the visible light used to irradiate a solution of the non-aggregating *cis*-isomers. This unprecedented photocontrol over supramolecular polymorphism highlights promising future prospects for similar pathway selection in supramolecularly polymorphic systems that consist of photo-controllable monomers.

## Data availability

All supporting data is provided in the ESI.†

## Author contributions

S. Y. and N. S. designed the project. N. S. performed most of the experimental works except for XRD measurement. S. Y. and N. S. prepared the overall manuscript, including figures. All authors, including T. S. and H. A., contributed by commenting on the manuscript. S. Y. supervised the overall research.

## Conflicts of interest

There are no conflicts to declare.

## Supplementary Material

SC-013-D2SC00690A-s001
