# Supplementary material for: Photo-modulation of supramolecular polymorphism in the self-assembly of a scissor-shaped azobenzene dyad into nanotoroids and fibers
Source: Chem Sci. 2022 Feb 23;13(11):3249–55. doi: 10.1039/d2sc00690a (PMC8926283; doi:10.1039/d2sc00690a)
Supplement: SC-013-D2SC00690A-s001 [file SC-013-D2SC00690A-s001.pdf]

Electronic Supplementary Material (ESI) for Chemical Science.
This journal is © The Royal Society of Chemistry 2022

Supporting Information

Photo-modulation of supramolecular polymorphism in the self-assembly of a scissor-shaped azobenzene dyad into nanotoroids and fibers

Natsuki Suda,[a] Takuho Saito,[a] Hironari Arima[a] and Shiki Yagai*[b,c]

[a] Division of Advanced Science and Engineering, Graduate School of Science and Engineering, Chiba University, 1-33 Yayoi-cho, Inage-ku, Chiba 263-8522, Japan.

[b] Department of Applied Chemistry and Biotechnology, Graduate School of Engineering, Chiba University, 1-33 Yayoi-cho, Inage-ku, Chiba 263-8522, Japan.

[c] Institute for Global Prominent Research (IGPR), Chiba University, 1-33 Yayoi-cho, Inage-ku, Chiba 263-8522, Japan.

Corresponding author:

Shiki Yagai; E-mail: yagai@faculty.chiba-u.jp

Table of Contents

1. General **S2**
2. Synthesis and Characterization **S4**
3. Supporting Figures **S8**
4. Supporting Reference **S14**

1. General

Materials and Methods

All reagents and solvents used in this study are commercially available reagent grade and used without further purification. Purification using column chromatography was performed using 63–210 μm silica gel. All the solvents for the preparation of the supramolecular assemblies were spectral grade and used without further purification. ^{1}H and ^{13}C NMR spectra were recorded on Bruker-AVANCE III-400M spectrometer and chemical shifts are reported in parts per million (ppm, δ) and are referenced to the signal of tetramethylsilane (TMS) at 0.00 ppm as internal standard. The resonance multiplicity is described as s (singlet), d (doublet), dd (double doublet), t (triplet), q (quartet), brs (broad singlet) and m (multiplet). ^{13}C NMR chemical shifts reported in δ (ppm) are referenced to the chemical shifts of $CDCl_3$ at 77.16 ppm. APCI-MS spectra were measured on an Exactive (Thermo Scientific). UV/Vis absorption spectra were recorded on JASCO V-760 and V-660 spectrophotometers equipped with JASCO ETCS-761 temperature controller. Circular dichroism (CD) spectra were recorded on JASCO J840 spectropolarimeter equipped with JASCO PTC-423L temperature controller. These spectra were recorded by using a screw-capped quartz cuvette of 1.0-mm optical pathlength. Fourier transform infrared (FT-IR) spectra were measured on JASCO FT/IR-4600 spectrometer. Dynamic light scattering (DLS) measurements were conducted on Zetasizer Nano (Malvern Instruments) using noninvasive backscattering (NIBS) technology under 4.0 mW He-Ne laser (λ = 633 nm). The scattering angle was set at 173°.

Atomic force microscopy (AFM): AFM images were acquired under ambient conditions using a Multimode 8 Nanoscope V (Bruker AXS) in ScanAsyst mode. SCANASYST-AIR (spring constant = 0.4 N/m; frequency = 70 kHz) or SCANASYST-AIR-HPI (spring constant = 0.25 N/m; frequency = 55 kHz) cantilevers were used. The AFM samples were prepared by spin coating aliquots of the aggregate solutions onto freshly cleaved highly oriented pyrolytic graphite (HOPG, 5 mm × 5 mm).

Scanning electron microscopy (SEM): SEM images were acquired on JSM-6510 scanning electron microscopy. The samples were prepared by drop-casting a suspension of the fibers onto a silicon substrate, dried under vacuum for 24 h, and then sputtered with Pt using JEOL JFC-1600 Auto Fine Coater before observation.

X-ray diffraction (XRD) analysis of precipitates: XRD patterns of bulk samples were measured using 0.154-nm X-ray (CuKα-beam) in the transparent mode using NANO-Viewer (Rigaku Corp.)

equipped with a Pilatus 100K (Dectris) detector. The scattering angle (2θ) and the position of the incident X-ray beam on the detectors were calibrated using several orders of layer reflections from silver behenate (d = 58.380 Å). The sample-to-detector distance was 7.6 cm. The obtained diffraction patterns were integrated along the Debye–Scherrer ring to afford 1D intensity data using 2DP software (Rigaku Corp.)

Photoirradiation Experiments: Photoirradiation experiments were performed using 365 nm UV light (LED lamp) and 470 nm visible light (LED lamp). The light intensity was calibrated off line by using an optical power meter (PM16-130, Thorlabs). Sample solution in a 1 mm-path length quartz cuvette or a glass vial was placed at the distance of 3 cm from the light source.

Preparation of pure nanotoroid solutions: A hot MCH solution of **1** (c_t = 500 μM) was quenched to 20 °C using an ice-bath and then allowed to stand at room temperature (*ca.* 26 °C). After 3 days, the resulting precipitates were removed by filtration using a membrane filter with a pore size of 450 nm to give a homogeneous solution of toroidal aggregates. The concentration (c_t) of this solution was estimated by UV/Vis absorption measurement and was adjusted to 100 μM by adding appropriate amount of pure MCH.

Estimation of the yield of toroidal aggregates at PSS_{Vis} solutions: Yield of the toroidal aggregates in PSS_{Vis} solutions was estimated based on the above-mentioned procedure. For this purpose, as-prepared PSS_{Vis} solutions at varying light intensity were allowed to stand for 3 days at room temperature to ensure all fibrous aggregates precipitated. To exclude the experimental error arising from the presence of small amount of *cis*-azobenzene-containing isomers, the absorption spectra were measured upon heating the solutions to 90 °C to estimate concentrations of monomers.

2. Synthesis and Characterization

Azobenzene dyad **1** was synthesized according to Scheme S1. Compound **4** was synthesized according to a reported procedure.[S1]

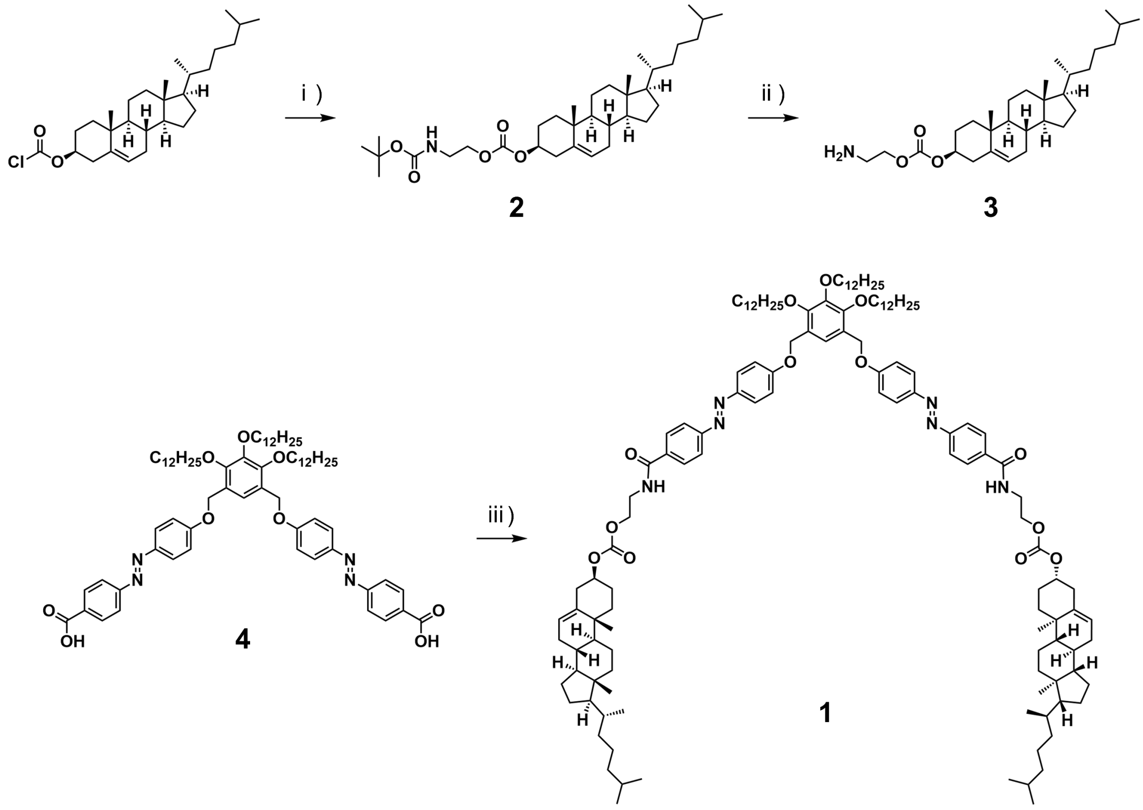

Scheme S1. Reagents and condition: i) 2-(*Tert*-butoxycarbonylamino)-1-ethanol, toluene, pyridine, r.t.; ii) Trifluoroacetic acid (TFA), **2**, CH_2Cl_2, r.t.; iii) 1-(3-Dimethylaminopropyl)-3-ethylcarbodiimide hydrochloride (EDC·HCl), *N*,*N*'-dimethyl-4-aminopyridine (DMAP), **3**, **4**, DMF, 0 °C → 80 °C.

Compound 2: 2-(*Tert*-butoxycarbonylamino)-1-ethanol (0.354 g, 2.20 mmol) was dissolved in a mixture of dry toluene (3 mL) and dry pyridine (0.521 g, 6.59 mmol), and the solution was stirred for 5 minutes at r.t.. After cooled the solution to 0 °C, cholesterol chloroformate (1.02 g, 2.27 mmol) dissolved in dry toluene (3 mL) was added dropwise, and the mixture was stirred for 17 h at r.t.. The solvent was evaporated to dryness. The residue was purified by column chromatography over silica gel (eluent: $CHCl_3$:methanol = 97:3) to give **4** as white solids (0.798 g, 63% yield).

^{1}H NMR (400 MHz, $CDCl_3$, 20 °C): δ = 5.40 (d, J = 5.2 Hz, 1H), 4.86 (brs, 1H), 4.53–4.46 (m, 1H), 4.18 (t, J = 5.1 Hz, 2H), 3.42 (q, J = 5.0 Hz, 2H), 2.43–2.37 (m, 2H), 2.03–1.78 (m, 6H), 1.70–1.48 (m, 8H), 1.44 (s, 9H), 1.40–1.04 (m, 12H), 1.01 (s, 3H), 0.91 (d, J = 6.6 Hz, 3H), 0.87 (d, J = 1.9 Hz,

3H), 0.86 (d, J = 1.8 Hz, 3H), 0.68 (s, 3H).

^{13}C NMR (100 MHz, $CDCl_3$, 20 °C): δ = 155.74, 154.36, 139.21, 123.09, 79.61, 78.13, 66.89, 56.68, 56.12, 49.97, 42.31, 39.70, 39.65, 39.52, 38.01, 36.83, 36.53, 36.18, 35.80, 31.90, 31.83, 28.37, 28.24, 28.03, 27.68, 24.28, 23.83, 22.85, 22.58, 21.04, 19.27, 18.72, 11.87.

HRMS (APCI): m/z calcd for $C_{33}H_{60}O_5N$ 574.4466 $[M+H]^+$, found 574.4467.

Compound 3: Compound **2** (0.277 g, 0.483 mmol) was dissolved in dry CH_2Cl_2 (2 mL) and the solution was stirred for 5 min at r.t.. To this solution, a mixture of TFA (0.824 g, 8.41 mmol) and dry CH_2Cl_2 (1.5 mL) was added slowly and the resulting mixture was stirred for 2.5 h at r.t.. The reaction mixture was evaporated to dryness to give **3** as white solids (0.276 g). This product was found to be almost pure and used for the next reaction without further purification.

^{1}H NMR (400 MHz, $CDCl_3$, 20 °C): δ = 8.26 (brs, 2H), 5.38 (d, J = 5.3 Hz, 1H), 4.47–4.44 (m, 1H), 4.38 (brs, 2H), 3.29 (m, 2H) 2.37–2.34 (m, 2H), 2.03–1.86 (m, 6H), 1.63–1.44 (m, 8H), 1.35–1.05 (m, 12H), 1.01 (s, 3H), 0.91 (d, J = 6.4 Hz, 3H), 0.87 (d, J = 1.8 Hz, 3H), 0.86 (d, J = 1.8 Hz, 3H), 0.68 (s, 3H).

^{13}C NMR (100 MHz, $CDCl_3$, 20 °C): δ = 154.02, 139.07, 123.16, 79.00, 63.61, 56.69, 56.21, 49.97, 42.32, 39.74, 39.52, 38.98, 37.77, 36.83, 36.49, 36.22, 36.18, 35.85, 31.91, 31.82, 28.26, 28.03, 27.44, 24.30, 23.93, 23.84, 22.85, 22.58, 21.06, 19.24, 18.74, 11.89.

HRMS (APCI): m/z calcd for $C_{30}H_{52}O_3N$ 474.3942 $[M+H]^+$, found 474.3943.

Compound 1: Compound **4** (64 mg, 0.056 mmol), compound **3** (68 mg, 0.14 mmol) and DMAP (25 mg, 0.21 mmol) were dissolved in 3 mL of DMF at 0 °C. To this mixture, EDC (36 mg, 0.19 mmol) was added and the mixture was stirred for 19 h at 80 °C. After cooling the solution to r.t., an excess amount of ethyl acetate was added to this reaction mixture and the solution became turbid. After stirring the suspension, precipitates thus formed were isolated by filtration. The resulting orange solid was dissolved in $CHCl_3$ and the solution was washed by water and then brine. The organic layer was dried over Na_2SO_4, filtrated and evaporated to dryness to give **1** as orange solids (29 mg, 25% yield).

^{1}H NMR (400 MHz, $CDCl_3$, 20 °C): δ = 7.88 (dd, J = 2.0, 9.1 Hz, 4H), 7.86 (s, 8H), 7.24 (s, 1H), 7.03 (dd, J = 2.1, 9.1 Hz, 4H), 6.71 (t, J = 5.5 Hz, 2H), 5.38 (m, 2H), 5.14 (s, 4H), 4.51–4.47 (m, 2H), 4.37 (t, J = 5.5 Hz, 4H), 4.10 (t, J = 6.6 Hz, 4H), 4.03 (t, J = 6.6 Hz, 2H), 3.80 (q, J = 4.7 Hz, 4H), 2.40–2.37 (m, 4H), 2.02–1.04 (m, 112H), 1.01 (s, 6H), 0.91 (d, J = 6.6 Hz, 6H), 0.88–0.84 (m, 21H), 0.68 (s, 6H).

^{13}C NMR (100 MHz, $CDCl_3$, 20 °C): δ = 167.08, 161.70, 154.67, 154.51, 151.97, 147.00, 145.80, 139.12, 135.15, 127.99, 125.26, 125.11, 123.19, 122.66, 115.04, 78.41, 74.28, 73.76, 66.52, 65.55,

56.67, 56.11, 49.96, 42.31, 39.70, 39.56, 39.52, 38.01, 36.82, 36.53, 36.18, 35.80, 31.96, 31.94, 31.90, 31.82, 30.43, 30.40, 29.77, 29.73, 29.68, 29.60, 29.55, 29.42, 29.39, 28.24, 28.04, 27.69, 26.20, 24.28, 23.84, 22.85, 22.73, 22.71, 22.59, 21.04, 19.27, 18.72, 14.16, 11.87.

HRMS (APCI): *m*/*z* calcd for $C_{130}H_{197}O_{13}N_6$ 2050.4933 $[M+H]^+$, found 2050.4956.

a)

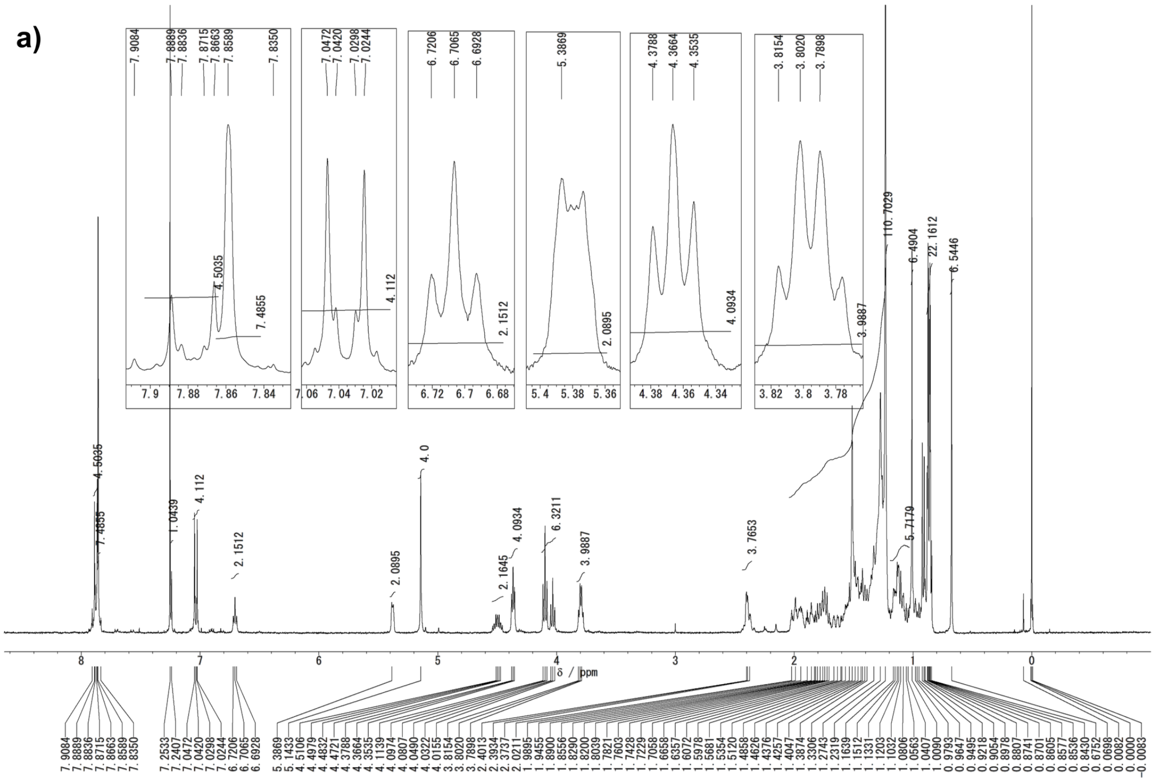

b)

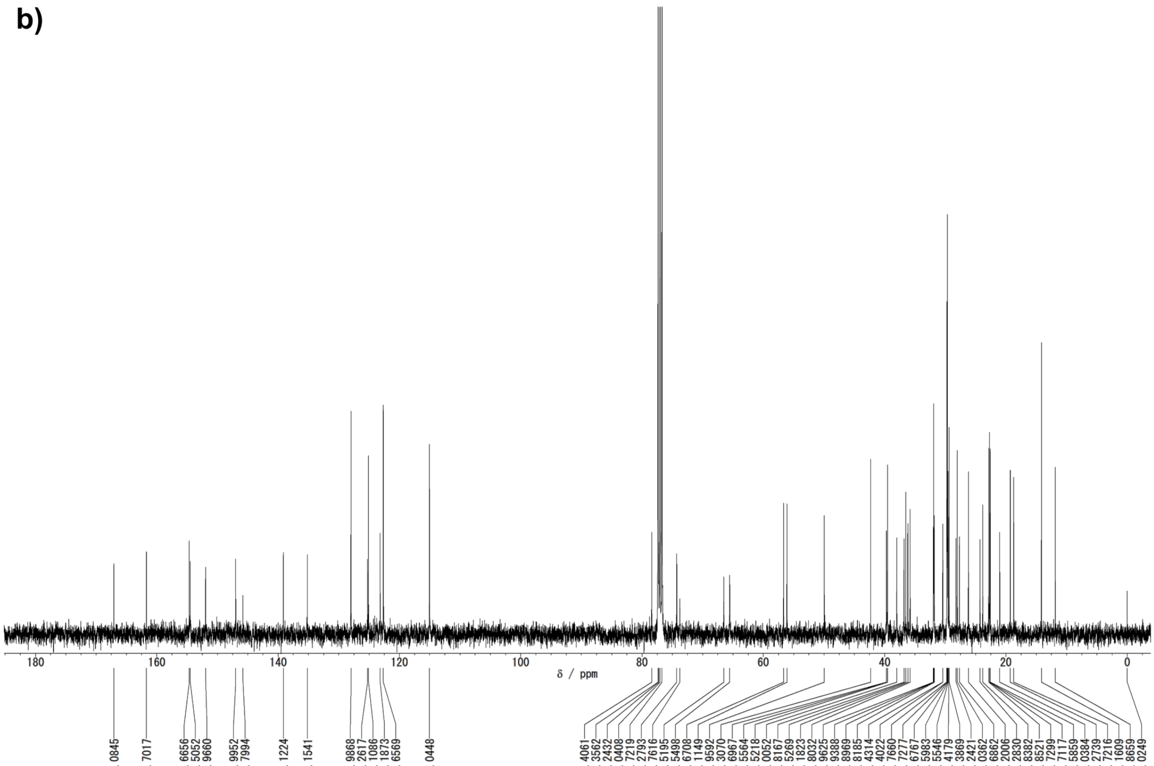

Chart S1. a) ^{1}H and b) ^{13}C NMR spectra of **1** in $CDCl_3$ at 20°C.

3. Supporting Figures

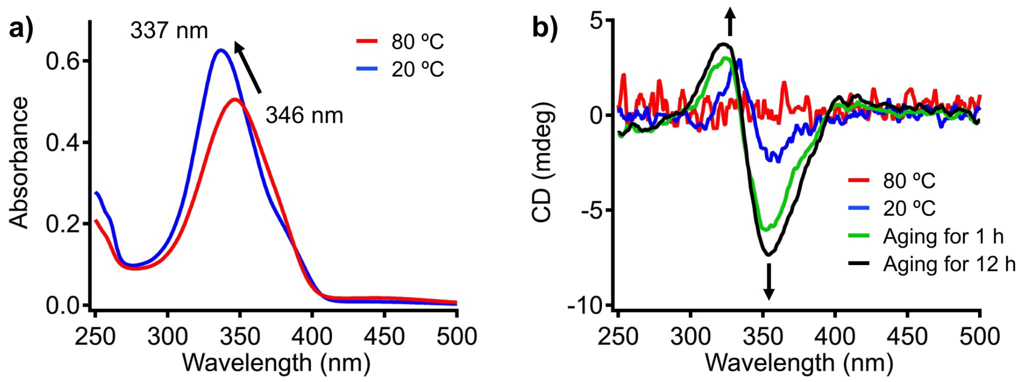

Fig. S1. a) UV/Vis absorption and b) CD spectra of **1** in MCH (c_t = 100 μM) at 80 °C (red lines) and 20 °C (blue lines) upon quenching. In b), the CD spectra upon aging the quenched solution for 1 h (green line) and 12 h (black line) are shown. These spectra are almost identical in shape with the CD spectrum of **1** in MCH upon cooling at a rate of 1 °C/min.

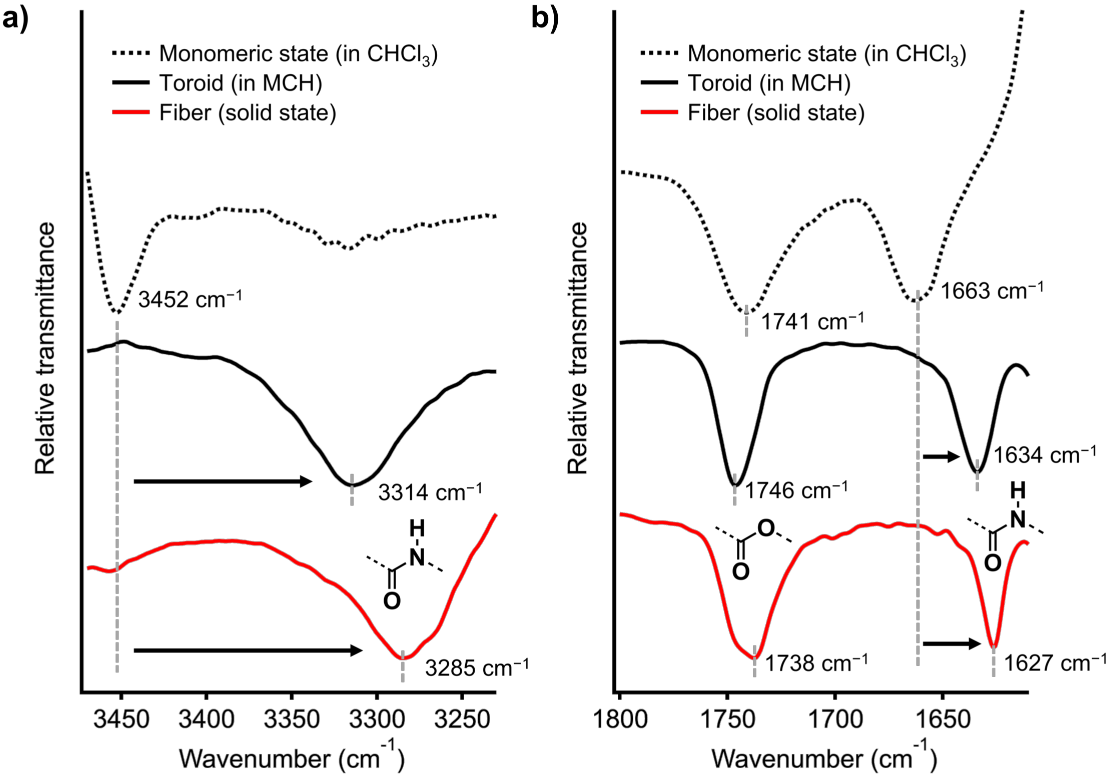

Fig. S2. FT-IR spectra of monomeric state of **1** in $CHCl_3$ (c_t = 500 μM, black dotted lines), nanotoroids of **1** in MCH (c_t = 100 μM, black solid lines) and fibers of **1** (solid state, red solid lines). The spectra display (a) the N-H stretching bands of the amide group and (b) the C=O stretching bands of the ester and amide groups.

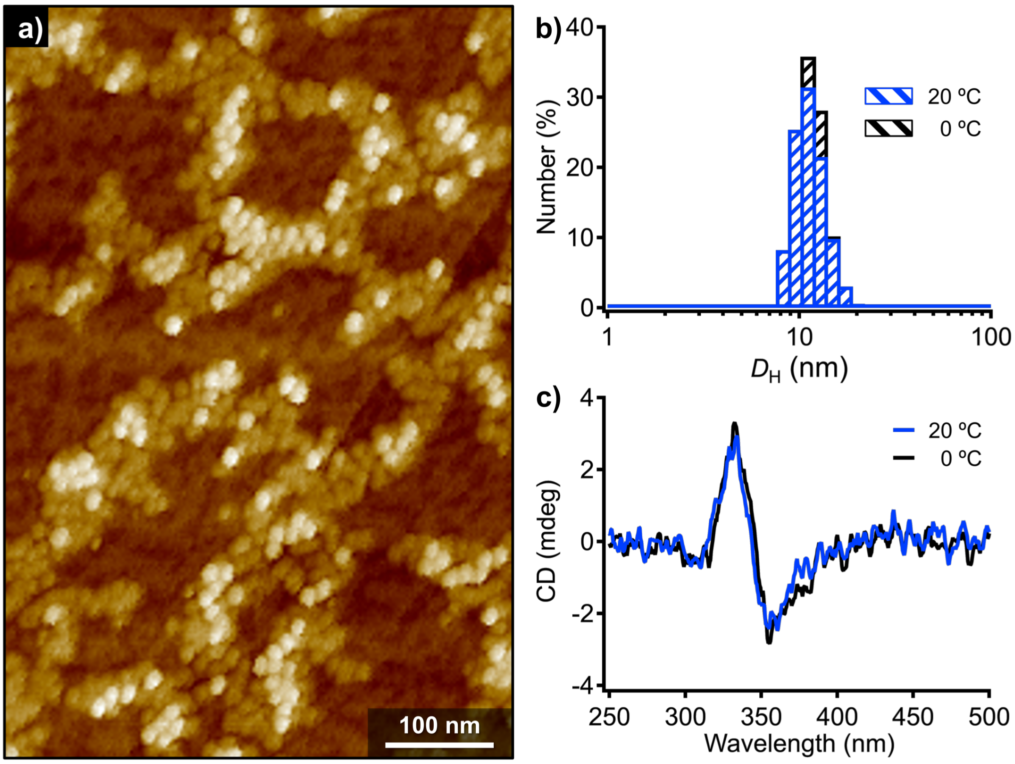

Fig. S3. a) AFM image of nanotoroids of **1** obtained from a MCH solution (c_t = 100 μM) prepared by quenching a hot monomeric solution to 0 °C. b) DLS size distribution of the quenched MCH solution of **1** at 20 °C (blue line) and at 0 °C (black line). c) CD spectra of the quenched MCH solution of **1** at 20 °C (blue line) and 0 °C (black line).

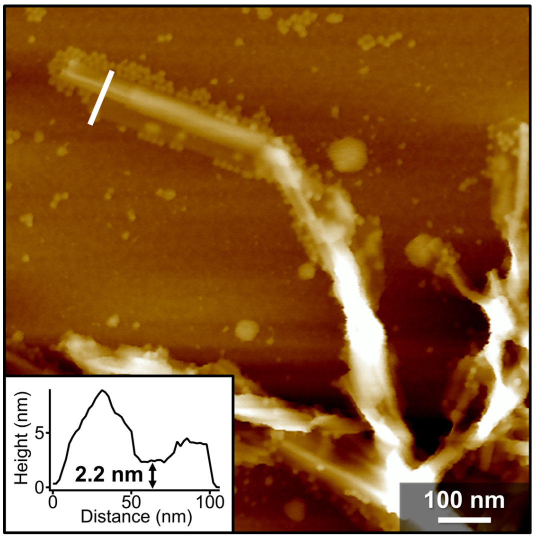

Fig. S4. AFM image of fibrous aggregates of **1** obtained by aging a quenched solution (c_t = 100 μM) for several hours at 20 °C. Inset shows an AFM cross-sectional analysis along the white line.

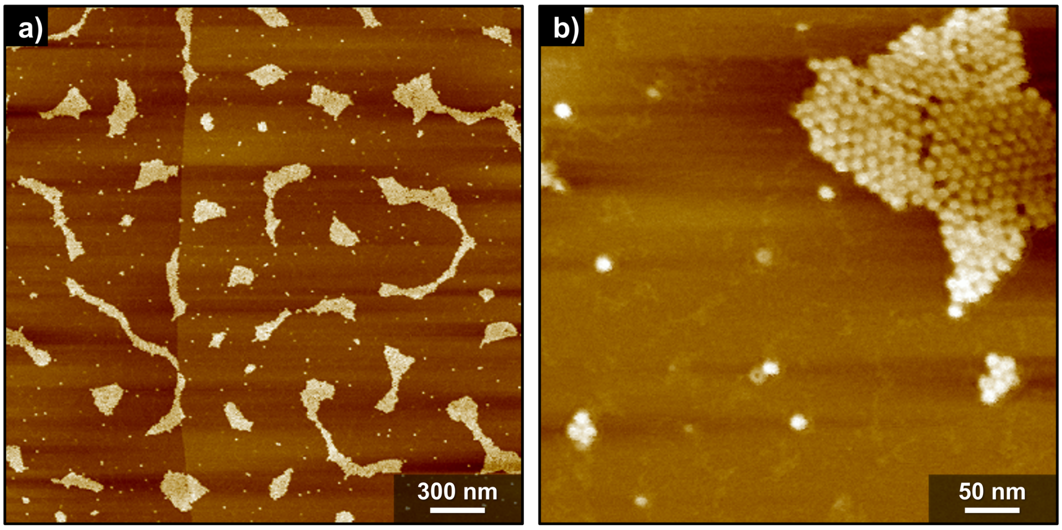

Fig. S5. a,b) AFM images of nanotoroids of **1** obtained after passing the precipitated solution of Figure S4 through a membrane filter.

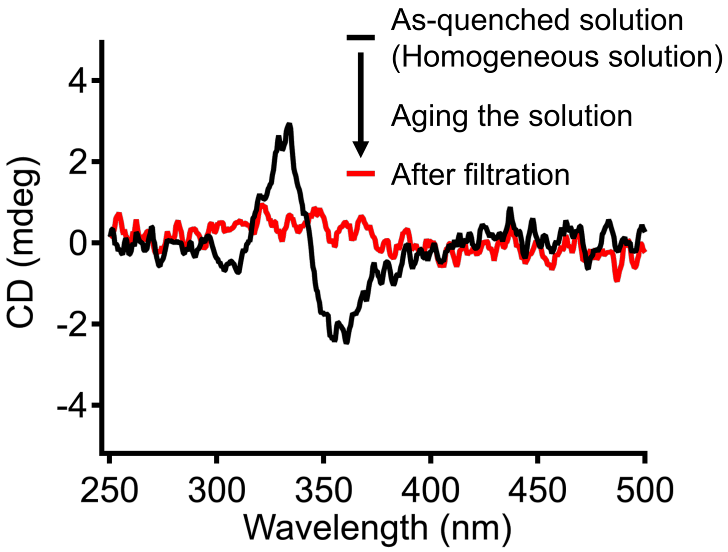

Fig. S6. CD spectra of a MCH solution of **1** (c_t = 100 μM) upon quenching a hot MCH solution before (black line) and after filtration (red line) using a membrane filter.

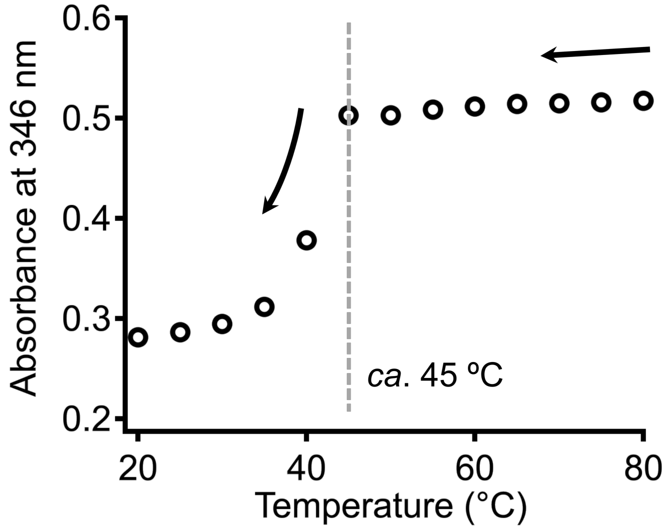

Fig. S7. Plot of the absorbance at λ = 346 nm versus temperature upon cooling a MCH solution of **1** (c_t = 100 μM). The absorption decreased drastically around 45 °C due to precipitation.

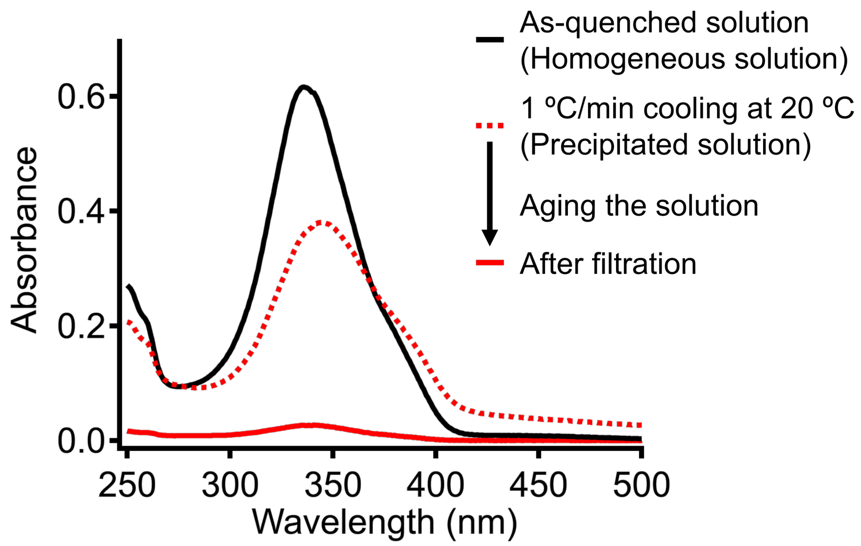

Fig. S8. UV/Vis absorption spectra of a quenched solution of **1** (c_t = 100 μM, black solid line), a suspension obtained by cooling a hot solution of **1** (c_t = 100 μM) at a rate of 1 °C/min (red dashed line), and the filtrate of the suspension obtained after filtration using a membrane filter (red solid line). The total concentration after filtration of the suspension was estimated to be 4 μM.

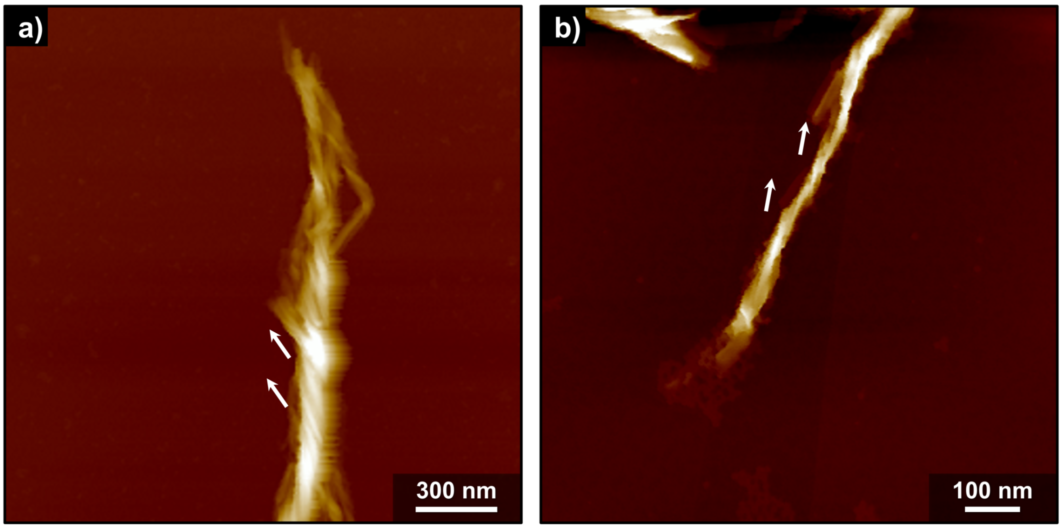

Fig. S9. a,b) AFM images of helical fibers of **1** obtained by cooling a hot MCH solution at a rate of 1 °C/min. White allows indicate left-handed helical structures.

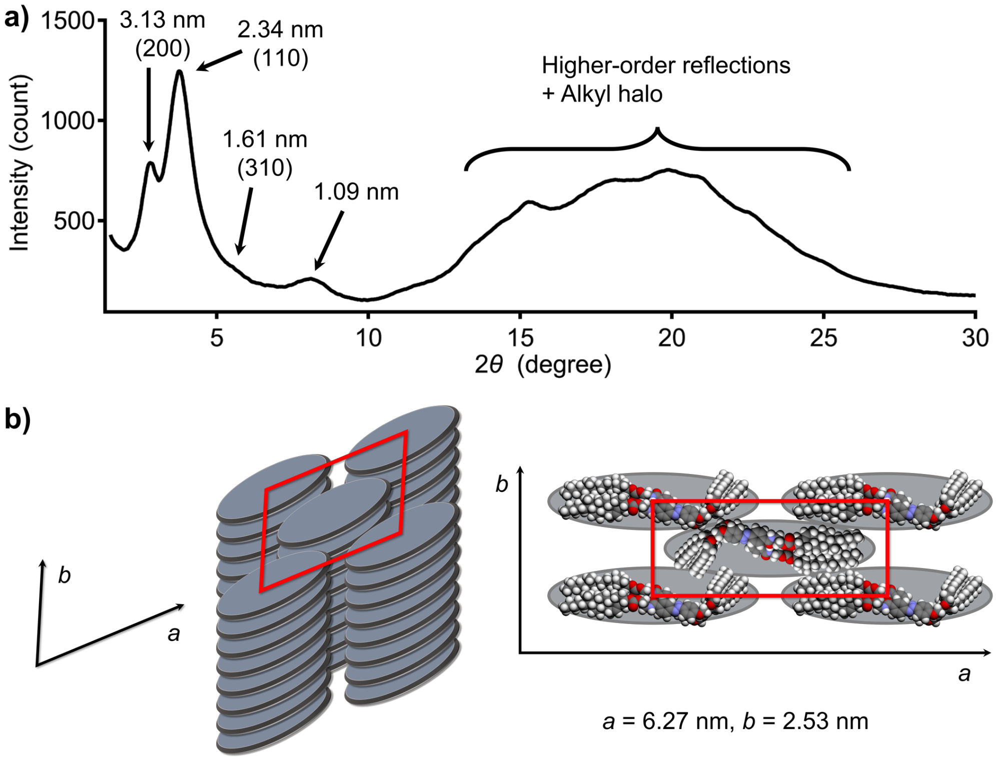

Fig. S10. a) XRD pattern of fibrous aggregates of **1**. b) Schematic representation of the proposed rectangular columnar packing structure of **1**.

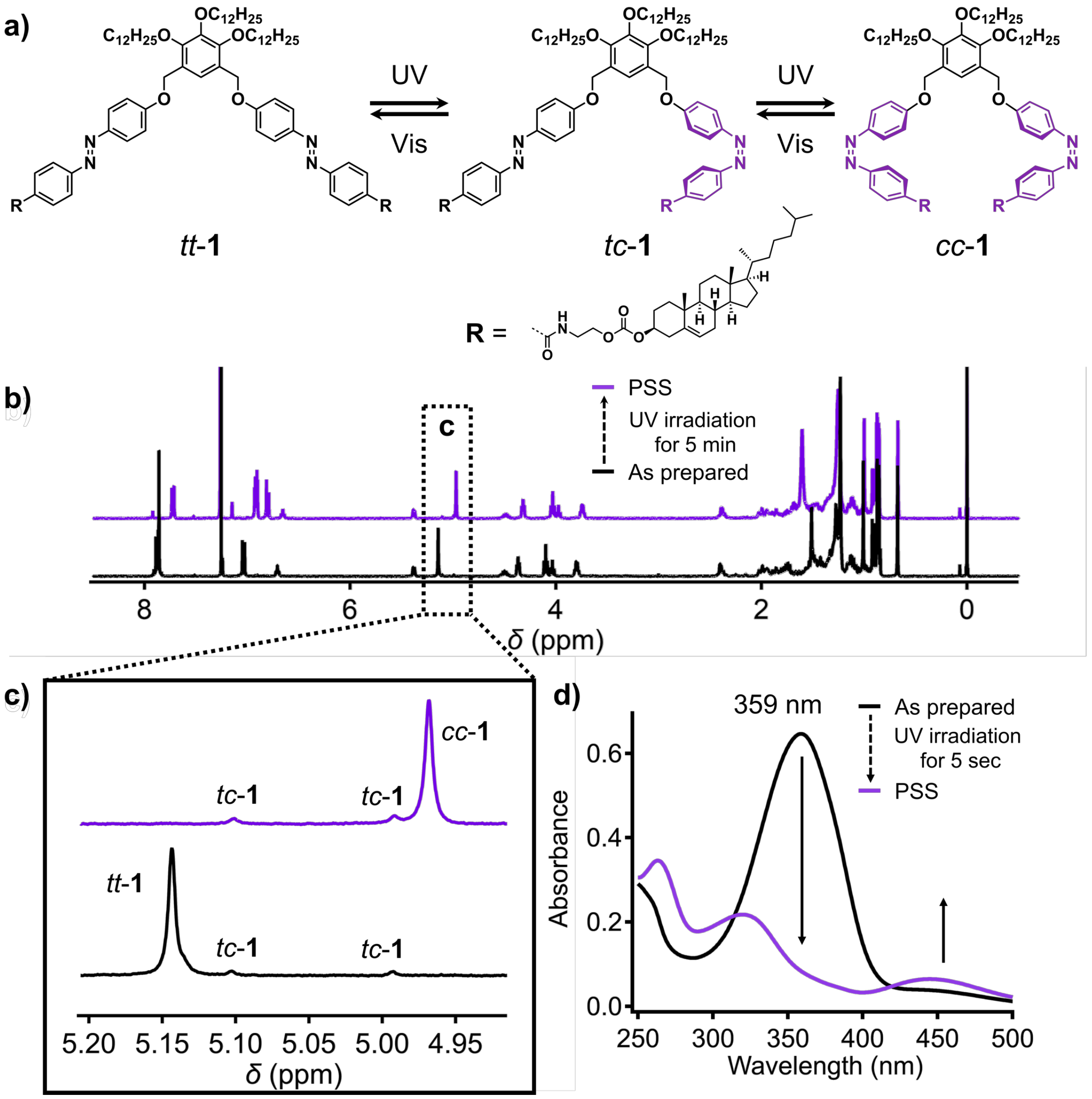

Fig. S11. a) Chemical structures of geometric isomers of **1** (*tt*-**1**, *tc*-**1** and *cc*-**1**, where *t* and *c* mean *trans*- and *cis*-azobenzene units, respectively). b) ^{1}H NMR spectra of monomeric **1** in $CDCl_3$ (c_t = 1.6 mM) at 20 °C before (black spectrum) and after irradiation with UV light (30 mW/cm^2) for 5 min to reach a PSS_{UV} (purple spectrum). c) Partial ^{1}H NMR spectra showing the benzylic proton signals of the three isomers. Integration of these signals revealed that the *tt*-**1**:*tc*-**1**:*cc*-**1** ratio changed from 94:6:0 (*trans*:*cis* = 97:3) to 0:12:88 (*trans*:*cis* = 6:94) at the PSS_{UV}. d) UV/Vis absorption spectra of pure *tt*-**1** in $CHCl_3$ (c_t = 100 µM) at 20 °C before (black spectrum) and after irradiation with UV light (30 mW/cm^2) for 5 sec (purple spectrum) to reach the PSS_{UV}. The absorption intensity at 359 nm decreased 87% upon irradiation with UV light, which corresponds to the 94% *trans*→*cis* isomerization according to the ^{1}H NMR analysis. This relationship has been used as a reference to calculate photoisomerization yield of azobenzene units in MCH.

4. Supporting Reference

S1 T. Saito and S. Yagai, *Org. Biomol. Chem.*, 2020, **18**, 3996–3999.
